# LINE-1 methylation in peripheral blood leukocytes and clinical characteristics and prognosis of prostate cancer patients

**DOI:** 10.18632/oncotarget.21511

**Published:** 2017-10-04

**Authors:** Yuyan Han, Junfeng Xu, Jeri Kim, Xifeng Wu, Jian Gu

**Affiliations:** ^1^ Department of Epidemiology, The University of Texas MD Anderson Cancer Center, Houston, Texas 77030, USA; ^2^ Department of Genitourinary Medical Oncology, The University of Texas MD Anderson Cancer Center, Houston, Texas 77030, USA

**Keywords:** LINE-1, methylation, prostate cancer, prognosis

## Abstract

Global DNA methylation of long interspersed nucleotide elements (LINE-1) in leukocytes has been suggested to be a risk factor for a few cancers. There has been no report of LINE-1 methylation in leukocytes as a risk factor for aggressive prostate cancer at diagnosis and prognosis after treatments. In this study, we measured the leukocyte DNA methylation of LINE-1 in 795 PCa patients and compared the methylation levels across different clinical subgroups. We then determined the association of LINE-1 methylation in leukocytes with clinicopathological variables at diagnosis using logistic regression analysis and biochemical recurrence in patients receiving active treatments (prostatectomy and radiotherapy) using Cox proportional hazard model after adjusting for age, BMI, smoking status, pack year, D’Amico risk groups, and treatments. Overall, the DNA methylation of LINE-1 was not associated with the risk of being diagnosed with high-risk prostate cancer or the risk of biochemical recurrence upon active treatments. Future studies are warranted to investigate other types of repetitive element methylation and longitudinal changes of global methylation in relation to prostate cancer risk and prognosis.

## INTRODUCTION

Prostate cancer (PCa) represents the most common cancer and the third leading cause of cancer death in American men [1]. Although the prognosis of PCa, particularly for locoregional PCa, has been excellent with a 5-year survival rate of 99%, overdiagnosis and overtreatment has become a rising clinical issue for PCa due to the limited ability of PSA screening to differentiate aggressive from indolent diseases [2]. Other clinical variables, such as tumor stage and Gleason Score (GS), are also not able to accurately distinguish aggressive and indolent PCa at diagnosis. Patients with similar clinical characteristics often have drastically different prognosis. Therefore, biomarkers are urgently needed to supplement clinical variables for differentiating aggressive from indolent PCa at diagnosis, allowing better-informed clinical management of localized PCa and avoiding unnecessary radical treatment.

Epigenetic changes including DNA methylation, histone modification and microRNA regulation play important roles in PCa development and progression [3]. DNA methylation is the best known epigenetic event. Hypermethylation of specific tumor suppressor genes and non-specific global hypomethylation frequently occur during tumorigenesis. Hypermethylation of the promoter regions of tumor suppressor genes leads to gene silencing whereas global hypomethylation is believed to cause genomic instability [4, 5]. A systemic review has shown that global hypomethylation in prostate tumor tissues is associated with PCa diagnosis (P<0.006) and prognosis (P<0.001) [6]. Recently, increasing studies measured global hypomethylation in peripheral blood leukocyte as surrogate genomic instability markers and reported significant associations of global DNA methylation with several cancers [7–14]. However, no study has evaluated the role of global leukocyte DNA methylation in the prognosis of localized PCa patients.

Global DNA methylation predominantly occurs within highly repetitive DNA sequences, such as long interspersed nucleotide elements (LINE-1) and short repetitive sequences Alu repeats [15, 16]. These elements are highly abundant and randomly distributed throughout the genome. The majority of published studies have used LINE-1 or Alu methylation as a surrogate marker for global DNA methylation [7–14]. In this current study, using a large PCa patient cohort, we measured LINE-1 methylation in peripheral blood leukocytes and analyzed its association with clinical characteristics at diagnosis and biochemical recurrence upon active treatment. To our knowledge, this is the first study to evaluate the association of global DNA methylation in leukocytes with the prognosis of PCa patients.

## RESULTS

The characteristics of the 795 PCa patients and the LINE-1 methylation level stratified by these characteristics are shown in Table 1. The majority of patients were either overweight (36.1%) or obese (31.7%). There were 353 (44.5%) never smokers, 364 (45.8%) former smokers, and 69 (8.7%) current smokers. Based on the biopsy before the treatment, 265 had GS = 6, 240 had GS=7 and 281 had GS ≥ 8. Using the D’Amico rick stratification criteria, 260, 217, and 313 patients were classified as low, intermediate, and high risk, respectively. Nearly 80% of the patients had PSA levels lower than 10 mg/ml, and about 10% each had PSA levels of 10 - 20 mg/ml and > 20 mg/ml. The overall methylation levels of LINE-1 across patients with different characteristics were similar and there was no significant difference across all strata (Table 1). We also compared the methylation level of each individual CpG site with GS (Table 2) and different D’Amico rick groups (data not shown). The methylation in the three CpG sites was not significantly different among patients with different aggressiveness at diagnosis. Logistic regression analyses did not show significant associations between LINE-1 methylation level and high GS or D’Amico risk group (data not shown). A subset of patients receiving prostatectomy had data on tumor size, surgical margin status, and lymph node invasion. None of these variables were associated with LINE-1 methylation (data not shown).

**Table 1:** LINE-1 methylation stratified by PCa patient characteristics

Characteristics	N (%)	LINE-1, β (SD)	*P* value
**Age at diagnosis, years**			
**<55**	66 (8.3)	79.4 (1.7)	Ref.
**55–65**	346 (43.5)	79.8 (1.9)	0.077
**>65**	383 (48.2)	79.8 (2.0)	0.105
**BMI at diagnosis, kg/m^2^**			
**<25**	115 (14.5)	79.9 (2.0)	Ref.
**25–29.99 (overweight)**	287 (36.1)	79.8 (2.0)	0.497
**≥30 (obese)**	252 (31.7)	79.6 (1.9)	0.153
**Unknown**	141 (17.7)	79.9 (1.9)	0.848
**Smoking status at diagnosis**			
**Non-smoker**	354 (44.5)	79.8 (1.9)	Ref.
**Former smoker**	364 (45.8)	79.8 (2.0)	0.648
**Current smoker**	69 (8.7)	79.3 (1.7)	0.036
**Unknown**	8 (1.0)	81.4 (1.1)	0.022
**D’Amico risk group**			
**Low**	260 (32.9)	79.7 (2.0)	Ref.
**Intermediate**	217 (27.5)	80.0 (2.0)	0.205
**High**	313 (39.6)	79.7 (1.9)	0.763
**Total Gleason score**			
**≤6**	274 (34.5)	79.7 (2.0)	Ref.
**7**	240 (30.2)	79.9 (1.9)	0.232
**≥8**	281 (35.3)	79.7 (1.9)	0.638
**Clinical tumor stage**			
**T1**	572 (71.9)	79.8 (1.9)	Ref.
**T2**	54 (6.8)	79.4 (2.2)	0.180
**T3–T4**	169 (21.3)	79.8 (2.1)	0.982
**PSA at diagnosis**			
**<10 ng/ml**	633 (79.6)	79.8 (1.9)	Ref.
**10–20 ng/ml**	80 (10.1)	79.7 (2.2)	0.847
**>20 ng/ml**	82 (10.3)	79.7 (1.8)	0.895
**Initial primary treatment**			
**Radical prostatectomy**	375 (47.2)	79.7 (1.9)	Ref.
**Radiotherapy**	133 (16.7)	79.9 (2.2)	0.460
**Surveillance or unknown**	268 (33.7)	79.7 (1.9)	0.936
**Other treatment**	19 (2.4)	80.0 (1.6)	0.578

**Table 2:** Methylation of LINE-1 and the aggressiveness of PCa at diagnosis

	GS 6		GS 7		GS 8		
CpG sites	Β	SD	β	SD	β	SD	P for Trend
**Site 1**	79.85	2.55	80.02	2.77	79.62	2.48	0.26
**Site 2**	81.8	2.19	82.03	1.81	81.89	1.95	0.58
**Site 3**	77.55	2.43	77.79	2.49	77.46	2.82	0.99
**Mean**	79.73	2.01	79.95	1.94	79.66	1.9	0.65

β: methylation index (%), SD: Standard Deviation.

We then analyzed the association of the methylation of these CpG sites with BCR in patients receiving active treatment (prostatectomy and radiotherapy) (Table 3). We dichotomized patients into high and low based on the medium methylation level. There were no significant associations with BCR for the overall methylation or any individual CpG site in this LINE-1 region. The HR for the association of overall LINE-1 methylation with BCR was 1.27 (95% CI, 0.80-2.03, p=0.31) after adjusting for age, BMI, smoking status, pack-year, D’Amico risk groups, and treatments.

**Table 3:** The association of LINE-1 methylation with biochemical recurrence (BCR) among localized prostate cancer patients who received active treatments

LINE-1 methylation	No BCR N (%)	BCR N (%)	Adjusted HR^a^ (95% CI)	*P* value
**Overall**				
Low	226 (83.39)	45 (16.61)	Reference	
High	202 (85.59)	34 (14.41)	1.27 (0.80-2.03)	0.310
**Site 1**				
Low	218 (83.85)	42 (16.15)	Reference	
High	210 (85.02)	37 (14.98)	1.16 (0.74-1.82)	0.514
**Site 2**				
Low	191 (82.68)	40 (17.32)	Reference	
High	237 (85.87)	39 (14.13)	0.97 (0.61-1.52)	0.881
**Site 3**				
Low	215 (83.33)	43 (16.67)	Reference	
High	213 (85.54)	36 (14.46)	1.07 (0.68-1.70)	0.772

^a^Adjusting for age, BMI, smoking status, pack-year, D’Amico risk groups, and primary treatment.

## DISCUSSION

DNA methylation plays important role in tumorigenesis and progression. Methylation profile is an inheritable feature that could affect genomic instability and gene expression. To our knowledge, this is the first study to evaluate the association of LINE-1 DNA methylation in peripheral blood leukocytes with the aggressiveness of prostate cancer. We did not find significant associations between LINE-1 methylation and presenting with high risk PCa at diagnosis or PCa prognosis.

LINE-1 is a highly abundant repetitive sequence in human genome with about half a million copies and accounting for nearly 18% of human genome [17]. The three sites we measured represent hundreds of thousands of same sequences in the genome, which is the reason that the methylation analysis of a short LINE-1 sequence can be used as an indicator of global DNA methylation. There have been many studies evaluating the associations between LINE-1 methylation in leukocytes and cancer risks and the results have been inconsistent mostly due to technical reproducibility and sample size issue [16, 18–24]. For prostate cancer, a previous large prospective, nested case-control study measured LINE-1 and Alu repetitive element methylation using the same pyrosequencing methods as ours in pre-diagnostic blood samples from approximated 700 pairs of prostate cancer cases controls nested in the Prostate, Lung, Colorectal, and Ovarian (PLCO) Cancer Screening Trial cohort, and did not find significant associations with prostate cancer [13]. Our study is the first one to report the association of LINE-1 methylation in leukocytes with the risk of aggressive prostate cancer at diagnosis and biochemical recurrence. Together with literature, our results indicate that the global methylation at LINE-1 region was neither a risk factor nor a prognostic factor for prostate cancer.

One interesting observation was that current smokers may have lower LINE-1 methylation than never smokers (P=0.036, Table 1). Consistent with this observation, several previous studies also showed that LINE-1 methylation was lower in current smokers than never smokers [25–27]. However, there were also studies showing lack of significant associations between LINE-1 methylation and smoking [28, 29]. Future studies are warranted to clarify the association between LINE-1 methylation and smoking as well as other epidemiologic variables.

Blood cell heterogeneity has been found to affect the DNA methylation measurements at specific locus [30, 31]. Previous studies have indicated that global DNA methylation is less likely to be affected by blood cell population as measured by pyrosequencing [32, 33]. A recent large study suggested that leukocyte composition has little confounding effect on association studies of leukocyte DNA methylation and several chronic diseases and risk factors [28]. Therefore, it is less likely that the lack of association between LINE-1 methylation and PCa aggressiveness in this current study was confounded by differences in blood cell subtypes among patients.

Our study has several strengths, including a relatively large patient population who were treated and followed-up at a single institution and a robust and reproducible pyrosequencing technique. There are a few limitations. First, we could not analyze the endpoint of mortality due to the small number of deaths of localized prostate cancer patients. Second, we only measured LINE-1 methylation. There are other types of repetitive elements and we cannot rule out that the methylation of other genomic regions may be associated with prostate cancer risk and prognosis. Third, we only collected one time blood at diagnosis and therefore could not determine longitudinal changes of LINE-1 methylation level. A recent study suggested that longitudinal changes of global methylation were associated with prostate cancer incidence and overall cancer mortality [14]. Future studies are warranted to determine whether longitudinal changes of global methylation are associated with the risk and prognosis of prostate cancer.

In summary, we have shown that global methylation of LINE-1 in leukocytes is not associated with prostate cancer aggressiveness or biochemical recurrence. Future studies are warranted to investigate other types of repetitive elements and longitudinal changes of global methylation in relation to prostate cancer risk and prognosis.

## MATERIALS AND METHODS

### Patients

A total of 795 non-Hispanic Caucasian men with histologically confirmed adenocarcinoma of prostate cancer from University of Texas MD Anderson Cancer Center were included in this study. Blood samples were collected before any treatment and genomic DNA was extracted and banked. Clinical and follow-up data were abstracted from patient medical records, which includes diagnosis date, performance status, clinical stage, histological grade and pathological stage, different treatment (including active surveillance, prostatectomy, radiotherapy, and hormone therapy), and progression (biochemical recurrence and metastasis). The MD Anderson Tumor Registry conducts annual vital status follow-ups for all cancer patients. Biochemical recurrence was defined as a serum PSA level of at least 0.2 ng/ml with a second confirmatory PSA level of at least 0.2 ng/ml for patients who undergo a radical prostatectomy [34] or with an increase in PSA level above 0.2 ng/ml and two consecutive increase over a minimum of three months for patients receiving external-beam radiotherapy [35]. We also tried other cutoff point of PSA, such as 0.4 ng/ml, as previously suggested to define BCR [36, 37] and the results were similar to the above criteria. This study was approved by the MD Anderson Cancer Center Institutional Review Board, and written consent forms were obtained from each patient.

### Methylation analysis with pyrosequencing

**Figure 1:**
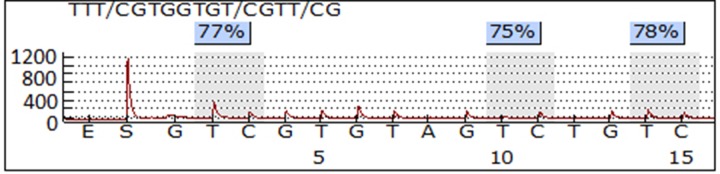
A representative pyrosequencing image of LINE-1 methylation assay The LINE-1 sequence contains three CpG sites. The frequency of methylation at each CpG site was calculated as the ratio of signal of C to the signal of T. and the overall methylation level of the sample was defined as the average of the three sites.

PCR and pyrosequencing of LINE-1 were performed as previously described [38]. In brief, 1 μg of genomic DNA was treated with sodium bisulfite using the EZ DNA Methylation-Gold Kit (Zymo Research, Irvine, CA) according to the manufacturer’s protocol. The samples were eluted in 20 μl of M-Elution Buffer and transferred to DNA Methylation Analysis Core at The University of Texas MD Anderson Cancer Center for subsequent PCR and pyrosequencing analysis. The assayed LINE-1 sequence was TTCGTGGTGCGTCG, which contained three CpG sites. Bisulfite-treatment of DNA converts all unmethylated cytosine to uracil, but leaves methylated cytosine intact. The ratio of the signal from cytosine (that signifies a methylated cytosine) to the sum of cytosine plus thymidine for each specific CpG site was termed β value as a parameter of methylation percentage at that site. The overall methylation was presented as the average methylation measured for all three sites. The PCR primer and pyrosequencing probe sequences were: TTTTGAGTTAGGTGTGGGATATA (forward), Bio-AAAATCAAAAAATTCCCTTTC (reverse), and AGTTAGGTGTGGGATATAGT [39]. PCR reactions were performed with ZymoTaq DNA Polymerase kit (Zymo Research, Irvine, CA) using 2 μl of bisulfite-treated DNA in a total volume of 15 μl, and the entire volume was used for each pyrosequencing reaction. Controls for high methylation (SssI-treated DNA), low methylation (WGA-amplified DNA) and no-DNA template were included in each run. In preparation for the pyrosequencing reaction, PCR product purification was done with streptavidin-sepharose high-performance beads (GE Healthcare Life Sciences, Piscataway, NJ), and co-denaturation of the biotinylated PCR products and sequencing primer (3.6 pmol/reaction) was conducted following the PSQ96 sample preparation guide. Pyrosequencing was performed on a PSQ HS 96 system (Biotage AB, Uppsala, Sweden) with the PyroMark Gold Q96 CDT Reagents (Qiagen, Hilden, Germany) according to the manufacturer’s instructions. The DNA methylation of each evaluated sample was determined as the average of the methylation of the three CpG sites in the assayed LINE-1 region, and each sample was run in duplicate. Figure 1 shows a representative pyrosequencing data of one sample.

### Statistical analysis

Similar number of GS 6, 7 and 8 cases was placed in each run and different batches were treated as a nuisance covariant in our analysis to control batch effect. We compared the methylation of three individual CpG sites and the average of the three CpG sites of LINE-1 among patients with different clinical characteristics at baseline using analysis of variance (ANOVA). Multivariate logistic regression was applied to evaluate the odds ratio (ORs) and 95% confidence intervals (CIs) for analyzing the association of LINE-1 methylation with aggressiveness of PCa at diagnosis after adjustment of co-variates such as age, smoking and BMI. We then analyzed the association of CpG site methylation with the risk of biochemical recurrence (BCR) in patients receiving radical prostatectomy or radiotherapy using multivariate Cox proportional hazards model adjusting for age, BMI, smoking status, pack year, D’Amico risk groups, and treatments. Time to BCR was calculated from the date of definitive treatment (radical prostatectomy or first chemotherapy) to the date of BCR or date of censor. The median follow-up time was 44.3 months.

## References

[1] Siegel RL, Miller KD, Jemal A (2017). Cancer Statistics, 2017. CA Cancer J Clin.

[2] Schroder FH, Hugosson J, Roobol MJ, Tammela TL, Ciatto S, Nelen V, Kwiatkowski M, Lujan M, Lilja H, Zappa M, Denis LJ, Recker F, Berenguer A (2009). Screening and prostate-cancer mortality in a randomized European study. N Engl J Med.

[3] Valdes-Mora F, Clark SJ (2015). Prostate cancer epigenetic biomarkers: next-generation technologies. Oncogene.

[4] Eden A, Gaudet F, Waghmare A, Jaenisch R (2003). Chromosomal instability and tumors promoted by DNA hypomethylation. Science.

[5] Breivik J, Gaudernack G (1999). Genomic instability, DNA methylation, and natural selection in colorectal carcinogenesis. Semin Cancer Biol.

[6] Zelic R, Fiano V, Grasso C, Zugna D, Pettersson A, Gillio-Tos A, Merletti F, Richiardi L (2015). Global DNA hypomethylation in prostate cancer development and progression: a systematic review. Prostate Cancer Prostatic Dis.

[7] Ardeljan D, Taylor MS, Ting DT, Burns KH (2017). The Human Long Interspersed Element-1 Retrotransposon: An Emerging Biomarker of Neoplasia. Clin Chem.

[8] Lou YT, Chen CW, Fan YC, Chang WC, Lu CY, Wu IC, Hsu WH, Huang CW, Wang JY (2014). LINE-1 Methylation Status Correlates Significantly to Post-Therapeutic Recurrence in Stage III Colon Cancer Patients Receiving FOLFOX-4 Adjuvant Chemotherapy. PLoS One.

[9] Harada K, Baba Y, Ishimoto T, Chikamoto A, Kosumi K, Hayashi H, Nitta H, Hashimoto D, Beppu T, Baba H (2015). LINE-1 methylation level and patient prognosis in a database of 208 hepatocellular carcinomas. Ann Surg Oncol.

[10] Li J, Huang Q, Zeng F, Li W, He Z, Chen W, Zhu W, Zhang B (2014). The prognostic value of global DNA hypomethylation in cancer: a meta-analysis. PLoS One.

[11] Park SY, Seo AN, Jung HY, Gwak JM, Jung N, Cho NY, Kang GH (2014). Alu and LINE-1 hypomethylation is associated with HER2 enriched subtype of breast cancer. PLoS One.

[12] Ikeda K, Shiraishi K, Eguchi A, Shibata H, Yoshimoto K, Mori T, Baba Y, Baba H, Suzuki M (2013). Long interspersed nucleotide element 1 hypomethylation is associated with poor prognosis of lung adenocarcinoma. Ann Thorac Surg.

[13] Barry KH, Moore LE, Liao LM, Huang WY, Andreotti G, Poulin M, Berndt SI (2015). Prospective study of DNA methylation at LINE-1 and Alu in peripheral blood and the risk of prostate cancer. Prostate.

[14] Joyce BT, Gao T, Zheng Y, Liu L, Zhang W, Dai Q, Shrubsole MJ, Hibler EA, Cristofanilli M, Zhang H, Yang H, Vokonas P, Cantone L (2016). Prospective changes in global DNA methylation and cancer incidence and mortality. Br J Cancer.

[15] Yang AS, Estecio MR, Doshi K, Kondo Y, Tajara EH, Issa JP (2004). A simple method for estimating global DNA methylation using bisulfite PCR of repetitive DNA elements. Nucleic Acids Res.

[16] Barchitta M, Quattrocchi A, Maugeri A, Vinciguerra M, Agodi A (2014). LINE-1 hypomethylation in blood and tissue samples as an epigenetic marker for cancer risk: a systematic review and meta-analysis. PLoS One.

[17] Rodic N, Burns KH (2013). Long interspersed element-1 (LINE-1): passenger or driver in human neoplasms?. PLoS Genet.

[18] Woo HD, Kim J (2012). Global DNA hypomethylation in peripheral blood leukocytes as a biomarker for cancer risk: a meta-analysis. PLoS One.

[19] Brennan K, Garcia-Closas M, Orr N, Fletcher O, Jones M, Ashworth A, Swerdlow A, Thorne H, Investigators KC, Riboli E, Vineis P, Dorronsoro M, Clavel-Chapelon F (2012). Intragenic ATM methylation in peripheral blood DNA as a biomarker of breast cancer risk. Cancer Res.

[20] Choi JY, James SR, Link PA, McCann SE, Hong CC, Davis W, Nesline MK, Ambrosone CB, Karpf AR (2009). Association between global DNA hypomethylation in leukocytes and risk of breast cancer. Carcinogenesis.

[21] Kitkumthorn N, Tuangsintanakul T, Rattanatanyong P, Tiwawech D, Mutirangura A (2012). LINE-1 methylation in the peripheral blood mononuclear cells of cancer patients. Clin Chim Acta.

[22] Liu J, Hesson LB, Meagher AP, Bourke MJ, Hawkins NJ, Rand KN, Molloy PL, Pimanda JE, Ward RL (2012). Relative distribution of folate species is associated with global DNA methylation in human colorectal mucosa. Cancer Prev Res (Phila).

[23] Xu X, Gammon MD, Hernandez-Vargas H, Herceg Z, Wetmur JG, Teitelbaum SL, Bradshaw PT, Neugut AI, Santella RM, Chen J (2012). DNA methylation in peripheral blood measured by LUMA is associated with breast cancer in a population-based study. FASEB J.

[24] Nan H, Giovannucci EL, Wu K, Selhub J, Paul L, Rosner B, Fuchs CS, Cho E (2013). Pre-diagnostic leukocyte genomic DNA methylation and the risk of colorectal cancer in women. PLoS One.

[25] Karami S, Andreotti G, Liao LM, Pfeiffer RM, Weinstein SJ, Purdue MP, Hofmann JN, Albanes D, Mannisto S, Moore LE (2015). LINE1 methylation levels in pre-diagnostic leukocyte DNA and future renal cell carcinoma risk. Epigenetics.

[26] Tajuddin SM, Amaral AF, Fernandez AF, Rodriguez-Rodero S, Rodriguez RM, Moore LE, Tardon A, Carrato A, Garcia-Closas M, Silverman DT, Jackson BP, Garcia-Closas R, Cook AL (2013). Genetic and non-genetic predictors of LINE-1 methylation in leukocyte DNA. Environ Health Perspect.

[27] Searles Nielsen S, Checkoway H, Butler RA, Nelson HH, Farin FM, Longstreth WT, Franklin GM, Swanson PD, Kelsey KT (2012). LINE-1 DNA methylation, smoking and risk of Parkinson’s disease. J Parkinsons Dis.

[28] Heiss JA, Brenner H (2017). Impact of confounding by leukocyte composition on associations of leukocyte DNA methylation with common risk factors. Epigenomics.

[29] Zhu ZZ, Hou L, Bollati V, Tarantini L, Marinelli B, Cantone L, Yang AS, Vokonas P, Lissowska J, Fustinoni S, Pesatori AC, Bonzini M, Apostoli P (2012). Predictors of global methylation levels in blood DNA of healthy subjects: a combined analysis. Int J Epidemiol.

[30] Reinius LE, Acevedo N, Joerink M, Pershagen G, Dahlen SE, Greco D, Soderhall C, Scheynius A, Kere J (2012). Differential DNA methylation in purified human blood cells: implications for cell lineage and studies on disease susceptibility. PLoS One.

[31] Adalsteinsson BT, Gudnason H, Aspelund T, Harris TB, Launer LJ, Eiriksdottir G, Smith AV, Gudnason V (2012). Heterogeneity in white blood cells has potential to confound DNA methylation measurements. PLoS One.

[32] Wu HC, Delgado-Cruzata L, Flom JD, Kappil M, Ferris JS, Liao Y, Santella RM, Terry MB (2011). Global methylation profiles in DNA from different blood cell types. Epigenetics.

[33] Bjornsson HT, Sigurdsson MI, Fallin MD, Irizarry RA, Aspelund T, Cui H, Yu W, Rongione MA, Ekstrom TJ, Harris TB, Launer LJ, Eiriksdottir G, Leppert MF (2008). Intra-individual change over time in DNA methylation with familial clustering. JAMA.

[34] Cookson MS, Aus G, Burnett AL, Canby-Hagino ED, D’Amico AV, Dmochowski RR, Eton DT, Forman JD, Goldenberg SL, Hernandez J, Higano CS, Kraus SR, Moul JW (2007). Variation in the definition of biochemical recurrence in patients treated for localized prostate cancer: the American Urological Association Prostate Guidelines for Localized Prostate Cancer Update Panel report and recommendations for a standard in the reporting of surgical outcomes. J Urol.

[35] Choo R, Danjoux C, Gardner S, Morton G, Szumacher E, Loblaw DA, Cheung P, Pearse M (2009). Efficacy of salvage radiotherapy plus 2-year androgen suppression for postradical prostatectomy patients with PSA relapse. Int J Radiat Oncol Biol Phys.

[36] Stephenson AJ, Kattan MW, Eastham JA, Dotan ZA, Bianco FJ, Lilja H, Scardino PT (2006). Defining biochemical recurrence of prostate cancer after radical prostatectomy: a proposal for a standardized definition. J Clin Oncol.

[37] Amling CL, Bergstralh EJ, Blute ML, Slezak JM, Zincke H (2001). Defining prostate specific antigen progression after radical prostatectomy: what is the most appropriate cut point?. J Urol.

[38] Ogino S, Kawasaki T, Nosho K, Ohnishi M, Suemoto Y, Kirkner GJ, Fuchs CS (2008). LINE-1 hypomethylation is inversely associated with microsatellite instability and CpG island methylator phenotype in colorectal cancer. Int J Cancer.

[39] Choi SH, Worswick S, Byun HM, Shear T, Soussa JC, Wolff EM, Douer D, Garcia-Manero G, Liang G, Yang AS (2009). Changes in DNA methylation of tandem DNA repeats are different from interspersed repeats in cancer. Int J Cancer.

